# Current Advances in the Management of Adult Craniopharyngiomas

**DOI:** 10.3390/curroncol29030138

**Published:** 2022-03-04

**Authors:** Montserrat Lara-Velazquez, Yusuf Mehkri, Eric Panther, Jairo Hernandez, Dinesh Rao, Peter Fiester, Raafat Makary, Michael Rutenberg, Daryoush Tavanaiepour, Gazanfar Rahmathulla

**Affiliations:** 1 Department of Neurosurgery, College of Medicine, University of Florida, 653 8th St W., Jacksonville, FL 32209, USA; mlaravelazquez@ufl.edu (M.L.-V.); yusuf.mehkri@neurosurgery.ufl.edu (Y.M.); ericpanther@ufl.edu (E.P.); hernandezjairo@ufl.edu (J.H.); daryoush.tavanaiepour@ufl.edu (D.T.); 2 Department of Neuroradiology, College of Medicine, University of Florida, 653 8th St W., Jacksonville, FL 32209, USA; dinesh.rao@jax.ufl.edu (D.R.); peter.fiester@jax.ufl.edu (P.F.); 3 Department of Pathology, College of Medicine, University of Florida, 653 8th St W., Jacksonville, FL 32209, USA; raafat.makary@jax.ufl.edu; 4 Department of Radiation Oncology, College of Medicine, University of Florida, 653 8th St W., Jacksonville, FL 32209, USA; michael.rutenberg@jax.ufl.edu

**Keywords:** craniopharyngiomas, sellar tumors, gross total resection, surgical resection, minimally invasive, neurological complications, radiation therapy, chemotherapy, molecular biology

## Abstract

Craniopharyngiomas (CPs) are slow growing, histologically benign intracranial tumors located in the sellar–suprasellar region. Although known to have low mortality, their location and relationship to the adjacent neural structures results in patients having significant neurologic, endocrine, and visual comorbidities. The invasive nature of this tumor makes complete resection a challenge and contributes to its recurrence. Additionally, these tumors are bimodally distributed, being treated with surgery, and are followed by other adjuncts, such as focused radiation therapy, e.g., Gamma knife. Advances in surgical techniques, imaging tools, and instrumentations have resulted in the evolution of surgery using endoscopic techniques, with residual components being treated by radiotherapy to target the residual tumor. Advances in molecular biology have elucidated the main pathways involved in tumor development and recurrence, but presently, no other treatments are offered to patients, besides surgery, radiation, and endocrine management, as the disease and tumor evolve. We review the contemporary management of these tumors, from the evolution of surgical treatments, utilizing standard open microscopic approaches to the more recent endoscopic surgery, and discuss the current recommendations for care of these patients. We discuss the developments in radiation therapy, such as radiosurgery, being used as treatment strategies for craniopharyngioma, highlighting their beneficial effects on tumor resections while decreasing the rates of adverse outcomes. We also outline the recent chemotherapy modalities, which help control tumor growth, and the immune landscape on craniopharyngiomas that allow the development of novel immunotherapies.

## 1. Introduction

CPs account for 1% of all primary intracerebral tumors in adults and up to 15% in children. These tumors show an incidence of 0.18 cases per 100,000 inhabitants each year [1,2,3]. The first description, by pathologist Jakob Erdheim [4] in 1904, described these as ‘hypophyseal duct tumors’, followed by Harvey Cushing, who described these tumors as CPs. CPs are benign tumors, classified as grade I by the World Health Organization (WHO), arising from the sellar–parasellar region [1]. Despite the histologically non-malignant nature of CPs complete resection is challenging due to the proximity and invasiveness into adjacent neural tissue, enveloping major vasculature, and involving the hypothalamus and pituitary [2,5,6]. Although survival at 5 years approximates 98%, there is a high rate of recurrence and post-surgical comorbidities that impair daily functions [3]. Despite that there are no distinctions in gender or race in the United States, a higher relative risk for craniopharyngioma in black patients has been described [7].

## 2. Clinical Manifestations

**Figure 1:**
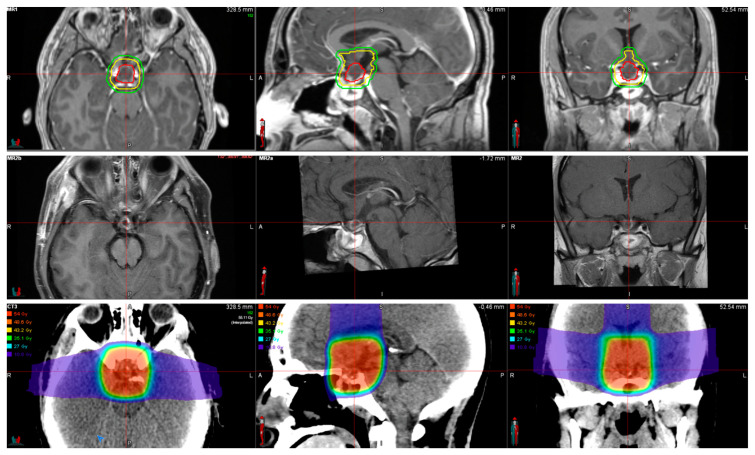
Twenty-nine year old patient with recurrent adamantinomatous craniopharyngioma treated with salvage surgery and post-operative proton radiotherapy. Row 1. Pre-op MRI shows suprasellar cystic tumor with enhancing wall involving the optic pathway. Pre- and post-operative tumor volumes inform the radiation target contours as overlayed on the images. GTV (red) = gross tumor volume (based on pre-op gross tumor), CTV (yellow) = clinical target volume (encompassing areas of tumor extent at initial presentation and areas at risk of subclinical disease), PTV (green) = planning target volume. Row 2. Post-op MRI shows residual cystic tumor involving the optic pathway. Row 3. Radiation planning CT with radiation dose overlay. Pencil beam scanning proton radiation was utilized to deliver a prescription dose of 54 GyRBE in 1.8 GyRBE fractions.

Clinically, patients with CPs present commonly with visual impairment (~80%), headaches (~60%), irregular menstrual periods (~60%), fatigue (~50%), gastrointestinal abnormalities (~30%), and weight disturbances (~20%) [8,9,10]. When the tumor obstructs the cerebrospinal fluid flow, hydrocephalus presents with headaches, progressing to further symptoms of raised ICP, and is one of the symptoms (along with lethargy, visual disturbances, papilledema, tumor calcification, and adhesiveness at surgery) proven to affect overall survival at 10 years, more often in children [11,12,13] (Figure 1). Additionally, the histological variant is also a determinant factor for survival. As confirmed by Wu et al. in a recent meta-analysis, adamantinomatous craniopharyngiomas (ACPs) have a higher risk of recurrence and poor prognosis than papillary craniopharyngiomas (PCPs), mainly due to their infiltrative nature and calcifications that limit a complete resection [14].

CP diagnosis is made by using contemporary imaging, such as c0omputed tomography (CT) and magnetic resonance imaging (MRI). For pediatric patients, a radiological classification preoperatively grading CP tumors based on the extent of hypothalamic involvement was developed by Puget et al. to help guide neurosurgeons on the best surgical strategies [15]. The classification demonstrates a significant relationship between the preoperative tumor grade according to hypothalamic involvement (grade 0 = none, grade 1 = affected but still visible hypothalamus, grade 2 = hypothalamic structures distorted) and the recommended surgical approach (GTR for grades 0 and 1, and STR for grade 2). However, in the adult population, no similar classification exists. When there is hypothalamic involvement, functional MRI (fMRI) is a helpful tool [16].

## 3. Histological Variants

**Figure 2:**
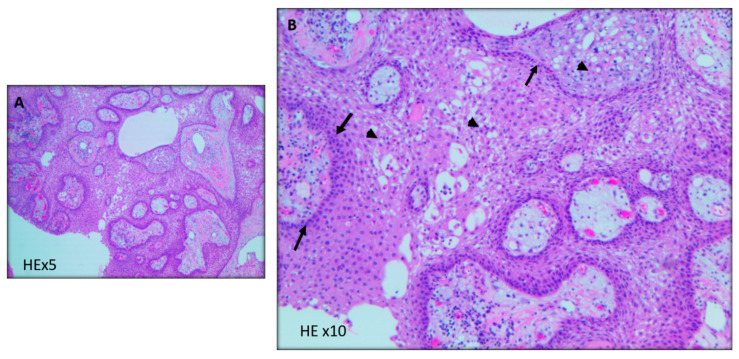
Craniopharyngioma with mixed papillary and adamantinomatous patterns with well differentiated squamous epithelium around fibrovascular cores. (**A**) Focal arears in the tumor show adamantinomatous pattern of craniopharyngioma displaying nodular and trabecular cellular growth with peripheral nuclear palisading ((**B**), arrow head) and looser plumper stellate reticulum cells ((**B**), arrows). HE = hematoxylin and eosin.

CPs are subdivided into two variants. ACPs are sellar tumors that show bimodal distribution, with one being in children around 5–15 years, and the other in adults at 45–60 years (less common) [5]. These not well-defined tumors are formed by a semisolid component (cyst and nodules) associated with fibrotic and hemorrhagic areas, along with some calcified areas [4]. The cyst can be uni- or multiseptated and filled with a liquid resembling “machine oil (brownish)”. CPs exhibit low mutation rates (~20 mutations per mB). However, ACPs show a higher prevalence rate in CTNNB1 gene mutations encoding β-catenin [17]. ACP diagnosis is usually confirmed after the multi-layer epithelium or the keratin nodules, along with nuclear immunoreactivity against β-catenin in a nodular array [18]. Due to the invasive nature of these tumors in surrounding structures, rates of morbidity are high, especially related to visual deficits and endocrine abnormalities due to hypothalamo-pituitary involvement [5]. Histologically, they are formed by three layers: (1) a peripheral palisading basal layer of squamous epithelial cells; (2) aggregates of stellate cells; and (3) a cyst-facing layer composed of flattened and keratinized squamous cells [5]. These tumors are accompanied by hemorrhagic changes with hemosiderin deposits, necrotic debris, inflammatory changes, cholesterol clefts, and glial reactive tissue [19] (Figure 2).

**Figure 3:**
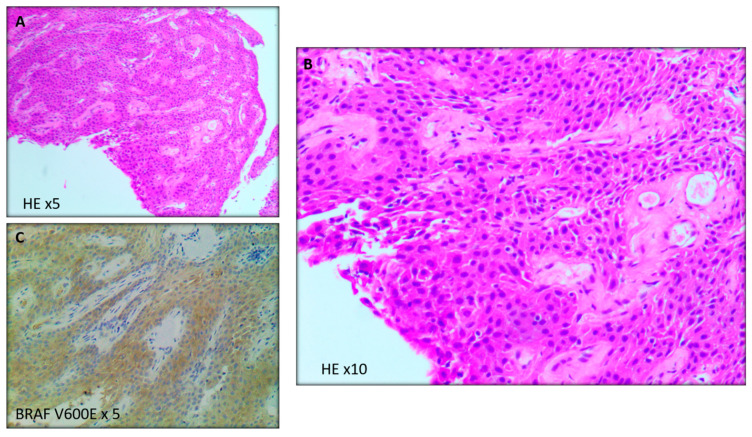
Tissue sections showing papillary configuration with cauliflower-like morphology of solid sheets of well-differentiated nonkeratinizing squamous epithelium around fibrovascular cores (**A**,**B**). Mitotic figures are rare to absent and no necrosis or significant nuclear atypia are seen. BRAF V600E immunostain is positive in tumor cells (**C**), supporting the diagnosis of papillary craniopharyngioma. HE = hematoxylin and eosin.

PCPs arise mainly in adults (15 to 50%), with a peak incidence at 40–50 years. PCPs are well-circumscribed lesions and less aggressive than ACPs, resulting in better survival at 5 years. PCPs harbor mutations in the BRAF gene (specifically p.BRAF-V600E) [20]. Microscopically, PCPs are solid tumors formed by squamous cells forming pseudo papillae mixed with fibrous and vascular stroma, with positive β-catenin immunoreactivity limited to the membrane (Figure 3) [20].

Tumor recurrence, however, does not depend on the histology variant [18].

## 4. Molecular Pathways Involved in Tumor Development

The pathogenesis of CPs includes several factors, some extrinsic, such as lifestyle, and others triggered directly by genetic and epigenetic changes of the person. As molecular medicine keeps advancing, some of the significant pathways that contribute to tumor initiation and progression have been discovered [21,22]. The main pathways associated with CPs include the wingless (Wnt)/β-catenin and the mitogen-activated protein kinases/extracellular signal-regulated kinase (MAPK/ERK). Both pathways are significant regulators of multiple biologic processes that, when disrupted, can contribute to tumorigenesis and have become significant targets for newly developed pharmacologic agents.

### 4.1. The Wingless (Wnt)/β-Catenin Pathway

The Wnt/β-catenin pathway is commonly involved in several processes during embryonic stages, including cell fate determination, organ development, cellular motility, and polarity and stem cell renewal [22,23]. Mutation of this canonical pathway have been allocated in cancer development and progression, with positive mutations (uncontrolled activation) held in colorectal (up to 70% of mutations), hepatocellular (25%), gastric (10–50%), endometrial (25%), and pancreatic (rare mutations) cancer [22,23,24,25]. In cancer, the persistent activation of the signal transducer Wnt causes a cytoplasmic accumulation of the protein β-catenin and its nuclear translocation, which coactivates transcriptional factors of the T-cell factor/lymphoid enhancing factor (TCF/LEF) family. The TCF/LEF transcription factors drive the expression of a subset of genes that produce different responses, with the main ones involved in cellular migration and proliferation [24,25,26,27]. Interestingly, the Wnt pathway shows a pleiotropic effect that regulates other non-fully understood signaling pathways such as GSK3 (to activate mTOR (another oncogenic pathway)); PA and CREB (involved in muscle development); and Ryk and Src (which regulate neuronal and axonal migration) [28]. Molecular investigations of craniopharyngiomas have revealed the role of this pathway in promoting neoplastic transformation, migration, and proliferation on the adamantinomatous subtype [29]. An aberrant activation on the β-catenin gene CTNNB1 is present in 80% of the adamantinomatous tumors (specifically in the exon 3) [27], which enhances the resistance of the protein to be degraded culminating in activation of the WNT/β-catenin pathway [30]. ACPs also show β-catenin nuclear aggregation (in up to 95% of the tumors) [30]. In addition, it was demonstrated that intranuclear accumulation of β-catenin has a correlation with epithelial transformation of adamantinomatous tumors, serving as a diagnostic molecular hallmark for this variant [27,31].

### 4.2. The Mitogen-Activated Protein Kinases/Extracellular Signal-Regulated Kinase (MAPK/ERK)

**Figure 4:**
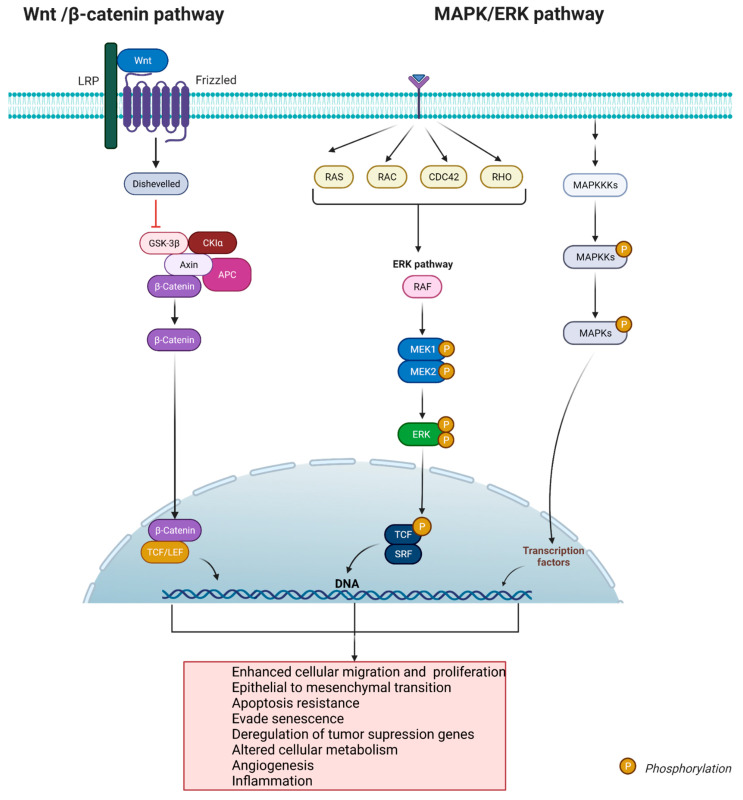
Representation of the molecular pathways involved in tumor formation and progression in CPs. The wingless (Wnt)/β-catenin and the mitogen-activated protein kinases/extracellular signal-regulated kinase (MAPK/ERK) pathways, are the main biological cascades involved in the development of the two histological variants of CPs: ACPs and PCPs. Created with Biorender.com (accessed on 28 November 2021).

The MAPK/ERK pathway is biologically involved in different processes including regulation of cellular proliferation, migration, differentiation, cellular growth, and apoptosis [32,33,34]. Altogether with JNK, p38 and BMK constitute the MAPK cascades. Aberrant activation of the MAPK/ERK intracellular cascade produces a gain-of function mutation that persistently transmits signals to small proteins, such as Ras and Raf [35,36]. The continuous stimuli of the kinases culminate in uncontrolled proliferation, altered apoptosis, enhanced migration, and modified cellular metabolism, all essential events for tumor formation and growth [37,38,39]. Oral, liver, pancreatic, endometrial, colorectal, renal, and brain cancer are some of the neoplasias described with enclosed mutations along the MAPK/ERK pathway [34,40,41,42,43]. In craniopharyngiomas, the MAPK/ERK pathway can be activated via mutations in the BRAF gene (as in the PCPs subtype), or by paracrine stimulation by secondary mediators such as interleukins and growth factors (as in the ACPs variant). Preclinical murine models along with human studies on craniopharyngioma have revealed a higher activation rate of the MAPK/ERK pathway on cancer cells positive to stem cell markers SOX2/SOX9, highlighting the importance of this pathway as an oncogenic driver [44].The BRAF gene mutation is present in 90% of the papillary variant and encodes a kinase with roles in cellular growth and differentiation, its mutation is present in 7% of different cancer types [45]. The mutation of the proto-oncogene BRAF is related with persistent activation of the MAPK/ERK pathway in cancer [46]. This finding opened the gate for the development and use of MAPK/ERK inhibitors in patients with this subtype with promising results (Figure 4) [46].

## 5. Current Treatment and Management for Craniopharyngiomas

### 5.1. Surgical Management

Surgical resection followed by radiotherapy is still considered the gold standard for craniopharyngiomas. Beyond providing rapid relief of symptoms, surgery allows tissue collection for histological diagnoses.

Although the goal for tumor resection is gross total resection, the extent of tumor resection is planned according to patient factors, tumor extent and invasiveness of adjacent neural and vascular structures, to avoid tumor recurrence [10]. Surgical subtotal resection (STR) is an alternative, and at times preferred, as 90% of progression-free survival at 5-years is achieved, with fewer comorbidities than with a complete resection [47]. There is a continued need for alternative treatments with increased efficacy and fewer adverse postoperative complications.

In a cohort treated between 1980 and 2009 in California, Schoenfeld and colleagues showed no significant changes in craniopharyngioma patients’ overall survival (OS) or progression-free survival (PFS) with gross total resection (GTR) when compared with STR and radiation (PFS; *p* = 0.544, OS; *p* = 0.735). They also showed that STR alone was associated with lower survival than GTR alone or STR plus radiation. In regards to comorbidities, GTR was associated with higher rates of neurologic (panhypopituitarism ~55% versus ~27%) and endocrine (diabetes mellitus ~57% versus ~14%) complications than STR [47]. Zacharia et al., by analyzing the surveillance, epidemiology, and end results (SEER) program database, showed better disease control with STR and radiation than GTR plus radiotherapy, in 644 patients analyzed between 2004 and 2008. Interestingly, they also showed higher incidence and worse control of the disease and survival in black patients than in the white population [7]. Sadashivam, in 2020, described similar long-term visual, endocrinological, and hypothalamic outcomes with subtotal resection and gross total excision in 95 adults operated between 2001 and 2013. When accompanied by radiation, they saw better tumor control in the STR group [48]. In recurrent tumors, repeated surgery should be considered with caution due to the high risk of complications and associated challenges for tumor identification [49,50].

When there is pituitary involvement, and the stalk is infiltrated by tumor cells, gross total resection is the best choice. In contrast, stalk preservation is preferred when not infiltrated by the tumor. Maintenance of the pituitary stalk does not change recurrence rates, but it decreases endocrine dysfunctions after surgery. Multiple studies in adult patients indicate that preservation of the pituitary stalk should be pursued as it decreases endocrine side effects. In children, complete resection of the gland with the stalk is preferred as the structure has no role in recovering endocrine functions. However, no established guidelines for either population exist [51,52]. Evolution of surgical techniques has resulted in better visualization, with endoscopic imaging and endoscopic endonasal surgery (EES) showing better outcomes than the conventional transcranial approach (TCA) due to tumor location, mainly in the sellar/suprasellar region. The endoscopic transsphenoidal approach, when compared to the interhemispheric one, achieves better gross total resection with less residual tumor while reducing morbidity and mortality [49,53,54]. This is under study in recurrent craniopharyngiomas by Li et al. [55]. Some scholars, however, have shown controversial findings in regard to EES. In a prospective study of 47 patients, Marx et al. found no differences in quality of life assessed with the anterior skull base quality of life questionnaire and olfactory function in EES vs. TCA. They described a higher incidence of CSF leaks in the EES group than TCA (29% vs. 15%) for the treatment of suprasellar craniopharyngiomas and reported higher visual outcomes and lower pituitary deficiencies in the same group [56]. Other studies have reported bleeding and pituitary abscesses as complications after EES [57].

**Figure 5:**
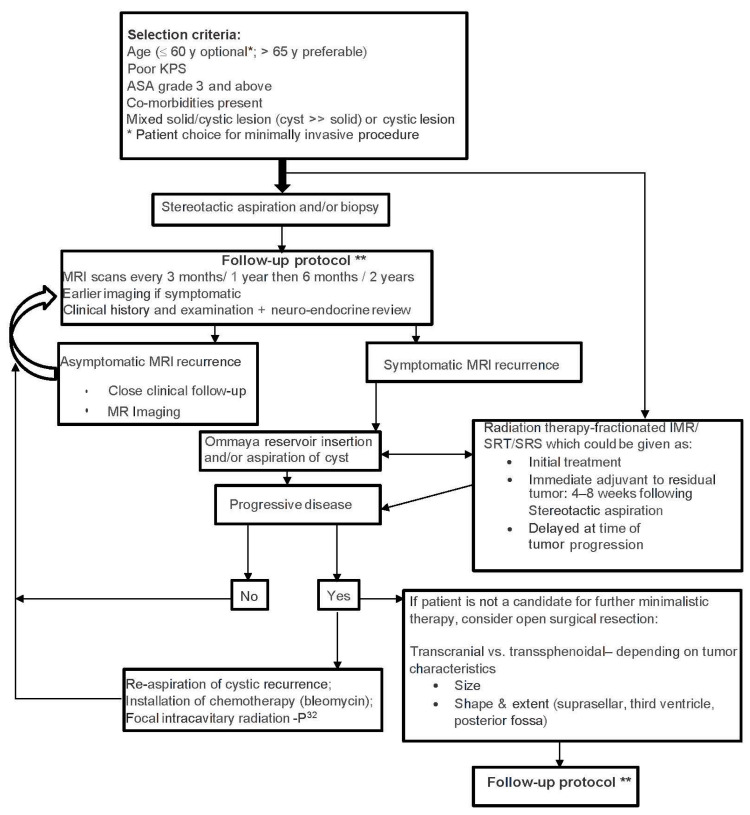
Algorithm by Gazanfar and Gene for the treatment of CPs with minimally invasive techniques combined with radiation. Taken without modifications from [58]. Abbreviations: KPS, Karnofsky Performance Status Scale; ASA, American Society of Anesthesiologists; MRI, magnetic resonance imaging; IMR, intensity modulated radiation; SRT, stereotactic radiation therapy; SRS, stereotactic radiosurgery. (published under Creative Commons Attribution License, which permits unrestricted use, distribution, and reproduction in any medium, provided the original author and source are credited). * Patient choice for minimally invasive produce. ** MRI scans every 3 months/1 year then 6 months/2 years, earlier imaging if symptomatic, clinical history, and examination + neuro-endocrine review.

In a case series of 11 patients, Rahmathulla and Barnett described the results of minimally invasive techniques, including burr hole aspiration, Ommaya reservoir placement, and ventriculoperitoneal shunting combined with Gamma knife stereotactic radiosurgery and intensity modulated radiotherapy (GKRS/IMRT)) for the treatment of CPs. They found better visual results and less complication rates post-surgery, with no morbidity–mortality rates peri-procedurally, along with shorter hospital stays. Their proposed algorithm for the treatment of CPs with these minimally invasive options is showed in Figure 5 [58].

Current studies about surgical approaches for CPs are included in Table 1.

### 5.2. Radiation Therapy

Radiation is an essential part of the treatment; the combined approach of surgery and radiotherapy improves tumor control than surgical resection alone. Recurrence-free survival at 10-years improves when radiotherapy is added to surgery (~90%), in comparison to GTR (~81%), or STR (~42%) alone. Radiation is also beneficial for recurrent tumors [64]. For parasellar tumors, radiation is usually administered at 45 to 60 Gray (2.0 Gray daily) 5–7 weeks (depending on each center); with an existing risk of radiation-induced toxicity [57,58,59,60,61,62,63,64,65,66,67,68,69,70].

Up-to-date radiation series studies for craniopharyngioma treatment are included in Table 2.

### 5.3. Stereotactic Radiosurgery

Stereotactic radiosurgery is a focused, non-invasive, image-guided type radiation therapy that utilizes convergent beams of high-energy x-rays, gamma rays, or protons to destroy abnormal tissue in a single radiation dose. LINAC and Gamma knife interventions present with similar clinical outcomes. LINAC however offers a wider range of treatment settings and a higher level of irradiation at a lower cost with the equipment being more widely available across the world in comparison to more expensive SRT modalities [76]. In SRS, multiple, intersecting beams allows for a high therapeutic dose in the treatment area, while surrounding tissue receives a relatively lower dose. Radiation-induced cell death of targeted abnormal tissue is the primary therapeutic pathway of SRS [77]. Recent improvements in SRS include more precise radiation delivery due to an improvement in tumor localization (using CT and MRI), as well as a reduction in radiation volume to healthy brain tissue due to an increasing number of beams during the procedure [78].

Stereotactic radiosurgery is mostly indicated for the treatment of “small discrete tumors” (mean tumor volume of 3 cm or less) as these tumors respond more quickly than larger ones [79].

The role of SRS in craniopharyngiomas is synergistic with the subtotal resection of the tumor. SRS can be a primary treatment but is usually applied after gross total or partial resection. Approximately 10% of totally resected craniopharyngiomas recur, and re-operation has shown increased morbidity and mortality. Additionally, complete resection is difficult to achieve due to the tumor’s proximity to critical structures [80]. The optic chiasm is a limiting structure for SRS, since this structure is capable of only receiving 8–10 Gy at once before optic neuropathy increases. The optic chiasm and nerves should be at least 3–5 mm away from the tumor for stereotactic radiosurgery to be recommended [81,82]. Yang et al., using SRS, showed that doses greater than 14.5 Gy were associated with longer progression-free survival [83]. The Shaw et al. study produced a five-year local control rate of 87% [84]. Pikis et al. showed a control rate of 91.6% (excluding cystic enlargement), although the median tumor size was only 1 cc, suggesting that benefits of SRS are mostly displayed in smaller tumors. It should be noted that since craniopharyngiomas show various degrees of solid and cystic compositions, the PSF listed by investigators is representative of the solid portion of the tumor, with cystic portions showing less response to radiotherapy [85]. Xu and colleagues demonstrated that a mixed or cystic tumor composition suggests a more unfavorable prognosis [86]. Due to the invasive nature of craniopharyngiomas and their proximity to optic structures, proper patient selection is imperative for the therapeutic success of SRS.

### 5.4. Fractionated Stereotactic Radiotherapy

Fractionated stereotactic radiotherapy (FSRT) utilizes the same stereotactic techniques as SRS, but the irradiation is distributed over multiple sessions. FSRT, similar to SRS, can be a primary therapy or adjuvant therapy after complete or partial tumor resection. Several authors have compared the timing of radiotherapy (primary treatment, right after resection, or after recurrence) with respect to progression-free survival and found no significant difference [87,88,89].

The reported local-control rates for FSRT are between 62 and 100% at 10 years, with the lower rates being attributed to a reduced irradiation dose [90]. One study showed overall tumor control of 81.3% for patients at 2, 5, and 10 years after LINAC-based FSRT of craniopharyngiomas, which shows very similar results as SRS. Currently, no significant differences in local tumor control between FSRT and SRS have been elucidated [91,92]. As previously discussed, SRS is indicated on craniopharyngiomas that are small and at least 3 mm away from the optic chiasm. The optic chiasm radiation tolerance to FSRT is 54 Gy/30 fractions, while the tolerance to SRS is 8–10 Gy. Multiple studies have shown reduced toxicity rates (nausea/vomiting, headache, neurocognitive and motor deficits, visual/hearing impairment) associated with FSRT compared to SRS, which is probably due to the minimized volume of irradiated tissue observed in FSRT [93,94]. The safety and excellent clinical outcomes of SRS and FSRT in the treatment of craniopharyngiomas is very well documented in the current literature, with the greatest indications for choosing a treatment being the radiation dose required to destroy the abnormal tissue, the size, number and location of the tumor, and the volume of healthy tissue that will receive the radiation dose (with FSRT having the greater flexibility) [95].

### 5.5. Intensity-Modulated Radiation Therapy

Intensity-modulated radiation therapy (IMRT) also uses high-energy photon and proton beams to irradiate abnormal tissue. The ability to manipulate the beams to conform to the shape of the tumor displays its distinct advantage. The radiation intensity of each beam is adjusted, and the targets of radiation change throughout the treatment [96]. The major advantage of IMRT is the decreased irradiation to healthy tissue surrounding the tumor [96,97,98,99]. IMRT is indicated for patients with tumors near critical structures since this technology has the potential of generating highly concave and conformal radiation distribution [100].

Similar to SRS and FSRT, studies have shown no variability in PFS or overall survival whether IMRT was delivered as adjuvant therapy or after remission in craniopharyngiomas. Studies also show that intensity-modulated radiation therapy has similar long-term clinical outcomes in craniopharyngiomas as 2D and non-IMRT 3D radiotherapy techniques, such as SRS and FSRT. Despite the reduced amount of radiation, IMRT patients still present with long-term toxicity, although some studies suggest that an increasing number of surgical interventions and the initial tumor volumes are the more likely offenders [101,102,103,104,105,106]. IMRT, despite still displaying long-term toxicity, may play a role in reducing radiation-induced complications later in the treatment, with more clinical data required to clearly define the long-term effectiveness and toxicity of this technique [107].

### 5.6. Proton Beam Therapy

Proton beam therapy (PBT) as a treatment for cancer has tremendously increased in popularity in the past few years. PBT uses a linear accelerator to generate concentrated beams of energy that are targeted at abnormal tissue. The unique advantage of this therapy lies in the physical properties of the proton beam, which lead to a relatively decreased scattered angle with a sharper dose distribution (with the highest dose point at the Bragg peak), and minimal to no exit radiation doses on healthy tissue [108]. Based on many in-vitro and animal studies, it has been assumed that protons have the same biological effects as photons, with 10% greater effectiveness.

Although SRS, FSRT, and IMRT have shown excellent clinical outcomes, the control for radiation-induced long-term toxicity remains suboptimal. Recent technological developments have allowed for expanded affordability and adoption of using PBT for CPs [109].

The use of PBT on craniopharyngiomas offers the same benefits evident in the treatment of other malignances and is clinically analogous to IMRT. Although IMRT and PBT have similar clinical outcomes, PBT has the potential to reduce brain and total body irradiation, therefore reducing the risk of complications and secondary cancer formation [110,111]. Luu et al. demonstrated that PBT offers a better opportunity for long term IQ retention in craniopharyngioma patients [112]. Intensity-modulated proton therapy (IMPT) is the most promising proton-beam therapy for craniopharyngiomas, in which proton “pencil-beams” of variable energy and intensity cover the target tissue. Costs of IMPT still remain about twice as high as IMRT, restricting its availability and research [113]. Still, several studies have noted a PBT 5-year local control rate of 85–100% for patients with craniopharyngioma, similar to radiation therapy [113,114]. Although PBT seems extremely promising for the reduction of radiation toxicity in patients undergoing treatment for craniopharyngioma, the uncertainty in the clinical dose due to tissue heterogeneity demands further investigation to justify the greater cost of treatment [114].

A summary of each radiotherapy modality is described in Table 3.

**Figure 6:**
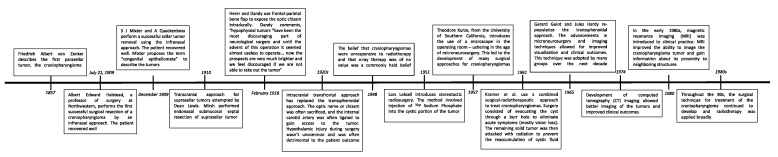
Timeline that includes the preceding events that lead the development of the surgical and radiation treatments of CPs [115,116,117,118,119].

Figure 6 depicts a timeline with the more important events that contributed to the history of surgical and radiation treatment of CPs.

## 6. Current Trends in the Treatment of Craniopharyngiomas

### 6.1. Brachytherapy

Brachytherapy (BT) is the direct addition of radioactive material into a tumor to control its growth and delay more aggressive treatment, such as resection and/or radiotherapy. BT has been used to treat tumors involved in prostate, breast, and gynecological cancers [120]. Recently, BT was shown to have promise in slowing the growth of craniopharyngiomas and sometimes results in complete elimination of the tumor and symptom resolution [121,122].

BT is indicated when a patient presents with a cystic craniopharyngioma as opposed to mixed and solid craniopharyngiomas [122]. Guimarães et al. performed a meta-analysis on 228 individuals who underwent BT for craniopharyngiomas [123]. The results of this study demonstrated that BT performed better in treating exclusively cystic craniopharyngiomas opposed to non-cystic craniopharyngiomas. BT was more effective in treating craniopharyngiomas in pediatric populations [123]. BT is used in pediatric presentations because aggressive resection may not be possible or desirable in young children, and BT has been shown to be effective in at least slowing the growth of the craniopharyngioma [121,122,123].

The radioactive element employed in craniopharyngioma BT is phosphorus-32 (P-32) [124,125,126]. The use of P-32 for craniopharyngioma treatment results in less radiation delivery to the patient, and it alone, or in conjunction with other treatments, can be successful in craniopharyngioma treatment [124]. Ansari et al. analyzed 9 patients who underwent craniopharyngioma resection with subsequent BT management but no radiotherapy [125]. The study showed that 5/9 patients underwent a follow-up surgery and 7/9 patients required radiotherapy to control the tumor [125]. Yu et al. reviewed 129 craniopharyngioma tumors treated with P-32 BT [121]. Of the tumors studied, “56 cysts (43.4%) showed resolution and/or nonrecurrence, which was classified as a complete response to treatment; 47 cysts (36.4%) showed a partial response” [121].

BT is a relatively non-invasive treatment that can be effective in treating craniopharyngiomas. The outcomes of BT are mixed, but most studies point to the use of BT for craniopharyngioma management following surgical resection [121,122,123,124,125]. Further studies are needed to evaluate BT effectiveness in treating craniopharyngiomas without any surgical intervention.

### 6.2. Chemotherapy

Chemotherapy is a widely used treatment for multiple types of cancers. Chemotherapy can be used preoperatively to shrink the tumor that will be resected postoperatively to destroy any remaining tumorigenic cells, or for recurrent tumors. Chemotherapy is performed in a variety of ways, including intravenously (IV), direct injection into the tumor, or orally. Chemotherapy is not a widely used treatment for craniopharyngiomas, but many studies have shown promising results by directly injecting the chemotherapy drugs bleomycin and IFN-α [126,127,128,129,130,131,132] into the intracranial tumor.

Bleomycin is a chemotherapy drug used for the treatment of cystic craniopharyngiomas. Because tumor resection is a procedure associated with high morbidity and mortality, many groups turned to the use of bleomycin to shrink intracranial tumors [130,131,132,133]. Though this drug has been shown to decrease tumor size in most patients, it has also been shown to have significant side effects leading to post-chemotherapy surgical resection. Hader et al. reviewed 7 patients who underwent intracystic bleomycin treatment and found that 4/7 patients had a tumor size reduction of more than 50%, 2/7 patients had tumor progression that led to surgical resection, and 1/7 patients underwent surgical resection due to persistent headaches [133]. One of the most reported side effects of bleomycin treatment is sudden onset cerebral edema [129,131]. Grob et al. reports a patient who was treated with intracystic bleomycin who developed “new edema involving the left pons, middle cerebellar peduncle, and anterior right cerebellar hemisphere”, which diminished after treatment was stopped [134]. Similarly, Hukin et al. reviewed a cohort of 17 patients who elected to undergo intracystic bleomycin treatment. Within this cohort of patients, 2/17 developed peritumoral edema [126].

IFN-α is a chemotherapeutic that is increasingly being used in the treatment of craniopharyngiomas [131,132,133,134]. IFN-α functions in activating the Fas apoptotic pathway in the cystic craniopharyngioma leading to reduced cyst volume [127,128,135]. Dastoli et al. reported 19 patients who underwent intracystic IFN-α injection and “11/19 patients had a reduction greater than 90%, 5/19 patients had a tumor reduction between 75 and 90%, and 3/19 patients had tumors reduced by less than 75%” [127]. Cavalheiro et al. treated 9 patients with intratumoral injection of IFN-α and 7/9 had complete tumor reduction and 2/9 had partial tumor reduction [128]. These results demonstrate the intriguing upside IFN-α treatment of craniopharyngiomas presents.

## 7. New Landscape in Craniopharyngioma Treatment: Immunotherapy

The role of the immune system in the pathogenesis of primary and recurrent craniopharyngioma has been under investigation for the past decade, yet much is still to be discovered. As will be discussed below, a majority of the literature has been conducted on ACPs and focuses on pediatric pathology.

Many potential immunotherapeutic targets are under investigation; however, none have entered clinical trials for the treatment of adult craniopharyngioma. Table 4.

### 7.1. The Immune and Inflammatory Components of Craniopharyngioma

**Figure 7:**
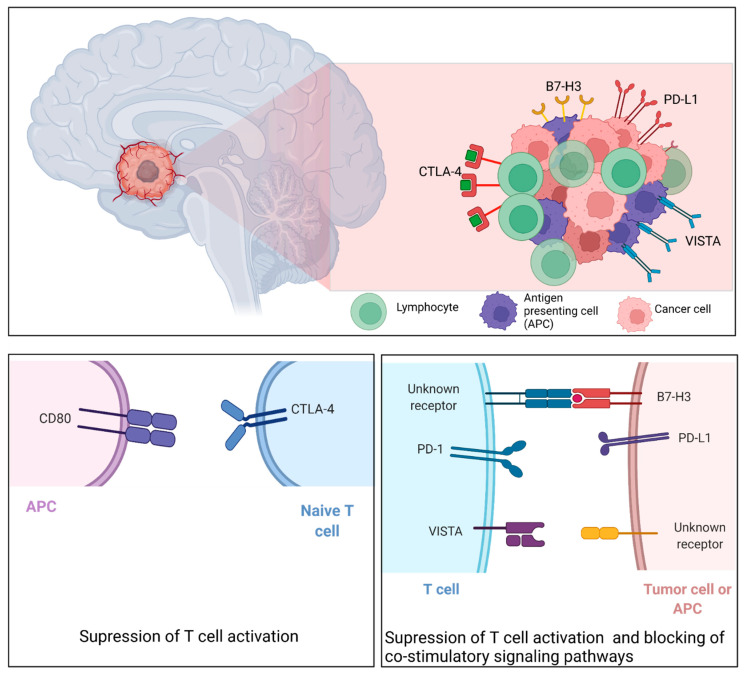
The study of the immune landscape of CPs allowed the development of novel immunotherapies for the treatment of these tumors. By targeting immunological checkpoints (CTLA-4, PD-L1, B7-H3, VISTA) the action of the immune system can be enhanced to specifically target cancer cells. Created with Biorender.com (accessed on 28 November 2021).

A link between the immune system and the proinflammatory response seen in craniopharyngioma pathophysiology was the first to be established. Mori et al. [139] conducted one of the first studies investigating the role of inflammation in ACPs pathogenesis and found highly elevated levels of cytokines, such as IL-6 associated with ACPs cyst generation. This was built upon by Pettorini et al. [19], who also found elevated levels of alpha-defensins 1–3, which are known to be associated with neutrophils. Interestingly, dense neutrophilic inflammation is commonly present in many PCPs [140]. Given that neutrophils can function as myeloid-derived suppressor cells, it is likely that the innate immune response plays a role in the pathogenesis of both ACPs and PCPs. In addition, multiple cytokine encoding genes that correlate with the infiltration of both myeloid and lymphoid derived cells have been shown to be significantly upregulated in ACPs [136]. The innate immune response also seems to be different between primary and recurrent craniopharyngioma. Lin et al. [141] found elevated levels of M2 macrophages—associated with tumor angiogenesis, progression, and worse prognosis [142,143]—in recurrent CPs. Interestingly, they also found an association between an increased number of M2 macrophages in primary CPs and risk of early recurrence (Figure 7).

### 7.2. Targeting B7-H3

B7-H3, an immune checkpoint from the B7 family, has been shown to be highly expressed and associated with poor prognosis in both ACPs and PCPs [138,140]. Its role as a key regulator of the immune microenvironment is related to suppression of T cell infiltration and blocking of co-stimulatory signaling pathways [19]. Interestingly, B7-H3 has also been associated with infiltration of IBA1+ cells, which, as mentioned earlier, contributes to tumor progression and recurrence [140,141]. B7-H3 would therefore serve as an effective therapeutic target for treatment of craniopharyngioma, especially in recurrence. The use of a B7-H3/CD3 bi-specific T cell engager has already shown therapeutic efficacy in a preclinical model by inhibiting cell growth [144]. Further research is warranted.

### 7.3. Targeting PD-L1

Elevated expression of PD-L1 has long been established in both ACPs and PCPs [139,142]. The spatial localization of PD-L1 differs between the two, with a preference for the fibrovascular core in PCPs and cystic lining in ACPs. In addition, PCPs tend to have higher overall expression of PD-L1 than ACPs and may respond better to anti-PD-L1 therapy [139,142]. Expression also seems to be associated with BRAF mutation, making combination therapy with a BRAF inhibitor an attractive potential therapy [138]. This is especially true in recurrent CPs, which has been shown to have higher levels of PD-L1 expression than primary CPs [138,139]. Although the mechanism underlying PD-L1 expression is largely unknown, it is thought to be specifically driven by BRAF V600E, specifically in PCPs [145,146]. This is due to an increase in T cell infiltration that led to tumor volume reduction following inhibition of BRAF/MEK, which is thought to be due to disruption of PD-L1 expression [146].

### 7.4. Targeting CTLA-4

To the best of our knowledge, no studies have specifically investigated CTLA-4 expression and blockade as related to craniopharyngioma. However, this may be a key therapeutic target for two reasons. First, combination checkpoint inhibitors have been shown to be effective at improving survival in multiple treatment-resistant cancers [147,148,149]. Aggressive tumors, such as craniopharyngiomas would benefit from such a strategy. In addition, CTLA-4 blockade has been shown to be effective in tumors with increased expression of immune related genes, such as craniopharyngiomas [150]. Given the likelihood of clinical response to a CTLA-4 inhibitor, there is a need for more research on therapeutic efficacy, especially in combination with other targeted therapies not directly involved with immune processes.

### 7.5. Targeting VISTA

One study investigated the role that VISTA, an immune checkpoint that suppresses T cell activation, plays in craniopharyngioma progression [144,150]. They found that VISTA expression was higher in PCPs and was correlated with patient age. This is expected, given that the PCPs subtype is more prevalent in adults. They also found VISTA expression to be associated with the BRAF mutation. Therefore, they suspect the RAS/RAF/MEK/ERK signaling pathway to be involved in VISTA expression. Modulation of this pathway, such as with an MEK inhibitor, provides an additional method for targeting this immune-active tumor.

## 8. Challenges for the Treatment of Craniopharyngiomas

### 8.1. Incomplete Resection

Craniopharyngiomas are often tightly associated with the hypothalamus and pituitary. As a result, complete resection is often avoided to protect vital postoperative hypothalamic function. Even with recent advances in imaging, surgical, and radiotherapy techniques, surgeons often choose incomplete resection despite high progression rates [151]. Recurrence rates have also been shown to be relatively high depending on the extent of resection [13,152,153]. The recurrence rate is the most important factor determining survival.

Although craniopharyngiomas are benign, they often invade nearby structures leading to the choice of a less aggressive surgical approach combined with radiotherapy [154]. Incomplete resection, however, can lead to postoperative challenges, such as cerebral spinal fluid leakage, hemorrhage, and hydrocephalus [155]. Revision surgery is possible, but the risks, which include further complications and death, outweigh the benefits [156].

### 8.2. Diabetes Insipidus (DI)

A frequent, unintended consequence of craniopharyngioma resection is disruption of the hypothalamic-pituitary axis. This disruption can lead to electrolyte imbalances causing a hyperosmolar extracellular state [152]. The result is an abnormally large amount of dilute urine excretion immediately post-surgery, leading to diabetes insipidus. Postoperative DI occurrence ranges from 7.5% to 54.2% [157,158]. The wide range in incidence is likely related to the variable diagnostic criteria for DI. Depending on the degree of fluid disturbance, the postoperative disruption may not be clinically recognized by all groups as DI [159,160]. This finding is more frequently reported in patients who underwent a transcranial resection of the craniopharyngioma as opposed to those who underwent an endoscopic, endonasal approach [161].

The most common form of postoperative diabetes insipidus is from disturbances in antidiuretic hormone (ADH) secretion from the posterior pituitary gland [162,163]. This form of DI is termed central diabetes insipidus (CDI) in contrast to nephrogenic diabetes insipidus (NDI) where ADH is present, but there is a lack of response from the kidneys [162]. Additionally, the postoperative CDI may be temporary or permanent, with a transient presentation of CDI being most common [161,162,163].

### 8.3. Metabolism and Hypothalamic Obesity

The hypothalamus is regarded as the central regulator of body weight [164,165]. Satiety and peripheral signals are regulated by the ventromedial nucleus and arcuate nucleus of the hypothalamus respectively [164]. One of the most common and debilitating complications from craniopharyngioma resection is hypothalamic obesity. A disruption in the endocrine pathway because of the tumor, surgical resection of the tumor, or radiation therapy can result in hypothalamic obesity [162]. In patients with hypothalamic obesity following craniopharyngioma resection, death from cardiovascular complications is 19-times higher than in the general population [166]. To compensate for reduced function of the hypothalamus, patients undergo life-long hormone replacement therapy to restore vital metabolic hormones. However, obesity is still present in 50–75% of craniopharyngioma patients [167].

Wu et al. showed that 49/120 (40.8%) experienced a 35% or greater weight gain within the first year after surgery, with an average weight gain of 17.59 ± 12.28% [168,169]. The most vulnerable among this group to weight gain were those with a lower preoperative BMI.

### 8.4. Visual Impairment/Loss

Because craniopharyngioma tumors typically lie near the optic chiasm, [170] preoperative visual defects are common. In most cases, surgical resection of the tumor results in visual restoration. However, Carnevale et al. showed that 21/1200 patients (1.75%) presenting with a craniopharyngioma had transient postoperative visual deterioration [170]. In this group, just 0.33% of patients were left with permanent visual deterioration.

El Beltagy et al. analyzed the postoperative outcomes of 65 patients with craniopharyngiomas. In the 16 patients with preoperative visual impairment, 15 patients had postoperative visual improvement [171].

### 8.5. Psychological Complications

The complex surgical approach to treating craniopharyngiomas leads to psychological and neurologic complications such as sleep disruption, impaired ability to concentrate, impulsivity, language disorders, and behavioral problems. These complications are more likely to occur if the tumor involves the hypothalamus [98]. Duff et al. performed a study examining the postoperative neurobehavioral outcomes of 121 patients. Among these patients, 27/121 (22.3%) experienced psychological problems to the point of needing therapeutic intervention [11].

Similarly, Rath et al. reported that the long-term cognitive impairment from craniopharyngioma resection caused just 40% of craniopharyngioma patients, diagnosed under the age of 10, to achieve adequate school and work attendance [77,78]. These effects caused poor social integration and financial dependence. Fjalldal et al. found significant differences (*p* < 0.05) in performance on verbal, memory, and attention and processing speed tests between patients and controls. Specifically, craniopharyngioma patients demonstrated delayed recall, a slower rate of learning, and worse fine motor skills [172].

## 9. Conclusions

Craniopharyngiomas are slow-growing tumors that need to be treated by a multidisciplinary team due to the long-term physical and psychological complications that can occur before and after their treatment. With the current advances achieved in molecular biology, the treatment of craniopharyngiomas will focus more on targeted treatment to decrease long-term sequelae. The current standard treatments of surgery and radiation are not appropriate solutions. With this updated review, we hope to provide clinicians with information about the multiple therapeutic options that have been (and are) under development to offer the best treatment for adult patients, especially since there is no official guideline for the treatment of these patients.

## Figures and Tables

**Table 1:** Clinical studies of open and endoscopic surgical approaches for CPs in the last 5 years.

Surgical/Endoscopic Recent Studies	Patient Population	Treatment	Summary of Report
Simonin 2020 [59]	16 patients, mean age = 42.9	Endonasal endoscopic approach	Endonasal endoscopic approach for the removal of suprasellar craniopharyngioma. Gross total resection was completed on 10/16 patients, with subtotal resection on the rest. Visual symptoms improved on 13/16 patients and remained unchanged for the rest. New endocrinological deficits were the most common complications (9/16), mostly diabetes insipidus. There was one mortality case and the mean follow-up time was 22.05 months, with 3/16 patients having a recurrence during that time.
Algattas 2020 [60]	62 patients, mean age = 41	Endonasal endoscopic approach	Retrospective analysis (2002–2015) of patients undergoing endonasal endoscopic approach for removal of craniopharyngioma. Gross total resection was initially achieved in 47% of cases, which increased to 77% by 2012. The review demonstrated similar outcomes between the present cohort and a transcranial approach. Although the literature suggests a greater gross total resection rate using a transcranial approach, studies have large variation. In this study, gross total resection and cerebrospinal fluid leak rates improved with time, suggesting there was a learning curve for complex resections in the institution.
Schelini 2019 [61]	20 patients, mean age = 7.5	Endoscopic endonasal transsphenoidal approach	Retrospective analysis (2007–2017) of patients with craniopharyngiomas. Gross total resection was achieved in 70% of patients and subtotal resection in 25% of patients. CSF leak occurred in 5% of patients and 55% of patients developed panhypopituitarism. Relapse occurred in 3/20 patients.
Santos de Oliveira 2017 [62]	8 patients, mean age = 10	Supraorbital eyebrow approach	Retrospective analysis (2014–2016) of patients who underwent supraorbital eyebrow approach. Incomplete resection took place in six patients and total resection took place in two patients. The author concludes that the supraorbital eyebrow approach offers sufficient working space for the surgical instruments and minimal surgical complications.
La Corte 2018 [63]	16 patients, mean age = 50	Endoscopic endonasal approach (*n* = 14), transcranial approach (*n* = 2)	Retrospective analysis (2005–2017) of patients with BRAF V600E mutant papillary craniopharyngiomas. A total of 68.7% developed postoperative diabetes insipidus and 56.3% increased their BMI. The authors concluded that patients with distinct BRAF V600E mutant papillary tumors may be treated with chemotherapy initially. However, if surgical intervention is necessary, the endonasal endoscopic technique should be favored over the transcranial approach.
Yamada 2018 [64]	65 patients, mean age = 9.6	Transsphenoidal approach	Retrospective analysis (1990–2015) of patients with childhood craniopharyngiomas. Gross total resection was achieved in 91% of the cases, and among this group, 12% had tumor recurrence. Vision improved in 62% of patients with pre-operative vision impairment and worsened in 11%. There were also six cases of CSF leak, three cases of meningitis, two cases of memory disturbance, and one case of hydrocephalus.
Patel 2017 [65]	16 patients, mean age = 11.0	Endoscopic transsphenoidal resection	Retrospective analysis (1995–2016) of patients with craniopharyngiomas. Gross total resection was achieved in 93.8%. A total of 66.7% of patients presented resolution of symptoms; vision improvements/retention were seen in 69.2% of patients. Postoperative complications included new-onset diabetes insipidus (46.7%), hypothalamic obesity (28.6%), panhypopituitarism (63.6%), and CSF leak (18.8%), and one intraventricular hemorrhage occurred. The author concludes that the endoscopic transsphenoidal approach can be used to achieve complete resection, but the hypothalamic-pituitary axis can be disturbed, and the CSF leak is a major postoperative complication.
Jamshidi 2018 [66]	28 patients, mean age = 19.3	Endoscopic endonasal approach	Retrospective analysis (2005–2017) of patients with craniopharyngiomas originating from the sellar inferior to the diaphragma sellae. Visual improvements were seen in 71% of patients with preoperative visual impairments. However, 21% of patients experienced iatrogenic complications, 7% experienced CSF leakage, and there was a recurrence rate of 18%. The author concluded that the transnasal approach can successfully treat subdiaphragmatic sellar tumors.
Forbes 2018 [67]	10 patients, (26–67 y/o)	Endoscopic endonasal approach	Retrospective analysis (2006–2017) of patients with craniopharyngiomas. Complete anterior pituitary insufficiency was seen in 90% postoperatively and complete posterior pituitary insufficiency was seen in 70% postoperatively. In 6 patients who had preoperative vision impairment, vision was normal in 4/6, postoperatively.
Alalade 2018 [68]	11 patients, mean age = 7.9	Endonasal endoscopic approach	Retrospective analysis (2007–2016) of patients with craniopharyngiomas in a variety of locations. Gross total resection was achieved in 45% of patients. Near-total resection was achieved in the remaining patients. Complications included anterior pituitary dysfunction (81.8%), diabetes insipidus (63.3%), and increased BMI (18%). Visual improvement was stable or improved in 73% of patients. The author concluded that the transsphenoidal approach is effective in removing craniopharyngiomas because it allows direct visualization of the hypothalamus, avoiding unnecessary injury.

**Table 2:** Clinical series using radiation modalities as treatment for CPs.

SRS and IMRT Recent Studies	Patient Population	Treatment	Summary of Report
Bidur 2017 [71]	25 patients, mean age = 30.12	Gross total resection or partial resection followed by radiotherapy (dose not specified).	A total of 21 patients had a gross total resection, with 4 patients having partial resection followed by radiotherapy. Out of the 21 patients who developed diabetes insipidus, 2 had partial resection followed by radiotherapy. In terms of quality of life, 2 patients died and 1 patient was dependent, all of which were part of the gross total resection group.
Ramanbhavana 2019 [72]	41 patients, mean age = 15.9	Gross total resection or partial resection followed by radiotherapy (dose not specified).	Epidemiological study and management of 41 craniopharyngioma patients. Patients who had surgical resection followed by radiosurgery (17/41) had better outcomes than surgery alone. Patients who were 18 years or older and those without a headache also had a better prognosis, although none of the comparisons were statistically significant.
Foran 2020 [73]	4 patients, ages 4, 14, 14, and 51	Recurrent craniopharyngioma treated with fractionated radiotherapy: RT1/RT2 dose (Gy)/fractions were 54/30 for three patients and 54/24 for 1 patient.	Retrospective study of 4 patients with recurrent craniopharyngioma, with a median follow-up of 33 months after reirradiation. A total of 3/4 patients had no further recurrences, and 1 patient developed progressive disease. In 3/4 patients, vision remained stable or improved after irradiation. None of the patients experienced new endocrine toxicities.
Lauretti 2017 [74]	10 patients, mean age = 43	Gross total resection or partial resection followed by radiotherapy (dose not specified).	Case series with systematic literature review of the neuroendoscopic treatment of cystic craniopharyngiomas. Case series yielded a recurrence rate of 20%, median PFS of 57 months, and no significant differences after using adjuvant radiotherapy. Authors suggest reserving radiotherapy for recurrent or progressive cases.
Rutenberg 2020 [75]	14 patients, ≥22 years old	All patients had gross disease at the time of radiotherapy, 54 GyRBE in 1.8 GyRBE/fraction; 9/15 patients had recurrent disease and the rest were de novo.	The three-year local control and survival was 100%. No radiotherapy-induced long-term visual disturbances. Ten patients experienced new endocrine deficits, including seven pan-hypopituitarism and eight diabetes insipidus cases.

**Table 3:** Radiotherapy modalities and mechanism of action.

Radiotherapy Modality	Mechanism
Stereotactic radiosurgery [76]	A single, high radiation dose is delivered using multiple, intersecting beams. Head frames or individual body molds are used to minimize movement.
Fractionated stereotactic radiotherapy [87]	Utilizes the same mechanism as stereotactic radiosurgery but distributes the radiation dose over multiple sessions to minimize toxicity to surrounding structures.
Intensity-modulated radiotherapy [96]	Multiple, intersecting beams are used to irradiate a target, but the intensity of each beam can be adjusted throughout the treatment.
Proton-beam therapy [108]	The physical properties of proton beams allow for a sharper dose distribution with minimal scattered radiation to healthy tissue. All previously listed delivery modalities can also be used for proton-beam therapy to further minimize toxicity.

**Table 4:** Clinical trials that have included immunological targets for the treatment of CPs.

Study	Model	Target	Proposed Mechanism	Potential Responders
Chen et al. 2019 [136]	Human primary craniopharyngioma cells	B7-H3	Increased T-cell and decreased IBA1+ (microglial) cell infiltration	ACP and PCP
Coy et al. 2018 [137]	Human primary craniopharyngioma cells	PD-L1	Inhibition of BRAF/MEK leading to increased T-cell infiltration	PCP and recurrent CP
N/A	Not directly investigated	CTLA-4	Increased efficacy when combined with an additional checkpoint inhibitor	ACP and PCP
Wang et al. 2020 [138]	Human primary craniopharyngioma cells	VISTA	Increased T-cell activation	PCP
